# Supplementary material for: Determinants of PrEP Uptake, Intention and Awareness in the Netherlands: A Socio-Spatial Analysis
Source: Int J Environ Res Public Health. 2022 Jul 20;19(14):8829. doi: 10.3390/ijerph19148829 (PMC9315833; doi:10.3390/ijerph19148829)
Supplement: Supplementary file 1 [file ijerph-19-08829-s001.zip › ijerph-1754986-supplementary.pdf]

Table S1. Metadata included in the modelling analysis

Outcome Variables	PrEP uptake	used to identify if samples in this study ever used PrEP in the past
	PrEP awareness	was operationalized as “having heard of PrEP” to identify if the samples were aware of the existence of PrEP
	PrEP intention	to identify whether MSM in the ? sample were willing to use PrEP if possible
Explanatory variable	Sociodemographic factors	
	Age	both continuous year and 10-year age bands were included
	Education level	which was categorised as low/median/low. Low education is defined as people who do not obtain a high school diploma, median education is defined as people who hold a high school diploma, and high education is defined as people who at least hold a Dutch applied science university (HBO) diploma.
	Employment status	which was categorised as employed/unemployed/retired/student

	Financial coping	which was categorised as (really) comfortable/neutral/(really) struggling. (really) comfortable is defined as those who living comfortably or really comfortably on present income; neutral is defined as those who neither comfortable nor struggling on present income; and (really) comfortable is defined as those who are struggling or really struggling on present income
	Sexual identity	which was categorised to homosexual/bisexual/straight or other
	Disclosure of sexual orientation (outness)	which was categorised to low/high. Low outness is defined as less than half people the participants were open about their sexual attraction with others; high outness is defined as more than half people the participants were open about their sexual attraction with others.
	Behavioural factors	
	Gay Intercourse recency	recency of having anal sex with another man, which was categorised to recent/more than 12 months ago

	Psycho-social factors	
	Safe_ efficacy_1 (Decision to have safe sex)	All men were asked if they agreed or disagreed with the following two statements: 'The sex I have is always as safe as I want it to be', which was translated to a recency from 1 to 5: 1 indicate strongly disagree to 5, which represents strongly agree.
	Safe_ efficacy_2 (Decision to reject sex)	All men were asked if they agreed or disagreed with the following two statements: 'I find it easy to say "no" to sex I don't want', which was translated to a recency from 1 to 5: 1 indicate strongly disagree to 5, which represents strongly agree.
	PrEP knowledge	operationalized as a set of 3 questions about the functionality of PrEP (how it works, and daily and on-demand regimen). It contained the number of PrEP knowledge already known among the sample, range from 0 to 3.
	HIV knowledge	operationalized as a set of 3 questions about the HIV transmission. It contained the number of

		HIV knowledge already known among the sample, range from 0 to 3
	PHQ-4 (Depression/anxiety level)	a 4-point item to reflect the level of depression or anxiety status of the participant, from 1 normal to 4 severe
	CAGE-4 (Alcohol dependency)	1 3-point item to reflect the level of alcohol dependency of the participant: 0 indicates missing measure, 1 indicates not alcohol dependent, and 2 indicates alcohol dependent.
	Relationship with HIV positive partner	which was categorised to no/yes with undetectable viral load/yes with detectable viral load/yes but do not know the viral load/I don't know.
	Condomless anal intercourse with non-steady partner	which was categorised to yes/no
	Condomless anal intercourse with steady partner	which was categorised to yes/no
	Condomless anal intercourse with	which was categorised to yes/no

	non-steady partner with diagnosed HIV	
	Ever diagnosed of syphilis, gonorrhoea, chlamydia	which was categorised to never diagnosed with any STI/ever diagnosed with any STI. Ever diagnosed with any STI is defined as ever diagnosed of the following infection in the past by the time of filling the survey: syphilis, gonorrhoea, chlamydia
	Transactional sex recency	is defined as ever buy/sell sex with/for money, which was categorised to never/ever but more than 12 months ago/ever within the last 12 months
	Injected drug using	is defined as ever injected steroid or non-steroid drugs, which was categorised to never/ever
	Chemsex recency	is defined as the recency of using an illicit recreational drug before or during the sex to improve sexual experience

Table S2. Sensitivity analysis of Estimated prevalence and standardised prevalence ratio, Randstad vs. the rest of the country (ROC), 2017 for descriptive analysis

Region		PrEP Use		PrEP awareness		PrEP intention	
		Prevalence (%, 95%CI)	SPR*	Prevalence (%, 95%CI)	SPR*	Prevalence (%, 95%CI)	SPR*
All	**Randstad**	7,91 (6,49 - 9,62)	1,41	90,84 (89,02 - 92,38)	1,11	46,26 (43,4 - 49,15)	1,02
	**ROC**	3,07 (2,41 - 3,91)	0,71	78,08 (76,23 - 79,83)	0,94	45,44 (43,31 - 47,58)	0,94
Without non-eligible men	**Randstad**	10.10 (8.27 - 12.25)	1,63	93.36 (91.51 - 94.83)	1,09	50.91 (47.61 - 54.20)	1,01
	**ROC**	3.96 (3.09 - 5.05)	0,64	80.06 (78.51 - 82.51)	0,94	50.00 (47.51 - 52.49)	0,99

Data are crude prevalence (95% confidence interval). Data are not age/education level/income status/employment status- standardised.

* Data are age/education level/income status/employment status- standardised. ROC=rest of the country.

Table S3. Sociodemographic status among the MSM study population by 10-year-age bands

		Age group details															
		16-19		20-29		30-39		40-49		50-59		60-69		70-79		80-89	
		n	%	n	%	n	%	n	%	n	%	n	%	n	%	n	%
Education level	High	22	1.1	405	19.8	424	20.7	508	24.9	465	22.8	179	8.8	38	1.9	3	0.2
	Median	35	4.6	173	22.5	130	16.9	150	19.5	190	24.7	71	9.2	20	2.6	1	0.1
	Low	48	11.5	51	12.2	64	15.3	69	16.5	113	27.0	55	13.2	17	4.1	1	0.2
Income status	High	54	2.5	341	15.6	420	19.2	520	23.8	571	26.1	223	10.2	51	2.3	4	0.2
	Median	42	5.4	217	27.9	139	17.8	155	19.9	144	18.5	60	7.7	21	2.7	1	0.1
	Low	9	3.4	71	26.4	59	21.9	52	19.3	53	19.7	22	8.2	3	1.1	0	0.0
Employment status	Employed	17	0.7	354	14.7	553	23.0	659	27.4	669	27.8	142	5.9	8	0.3	1	0.0
	Unemployed	4	1.3	53	17.4	47	15.4	65	21.3	87	28.5	47	15.4	2	0.7	0	0.0
	Retired	0	0.0	0	0.0	0	0.0	1	0.5	11	5.6	114	58.5	65	33.3	4	2.1
	Student	82	25.7	220	69.0	17	5.3	0	0.0	0	0.0	0	0.0	0	0.0	0	0.0

Data are n/N with %. Data are age standardised.

Figure S1 Performance check of the final model

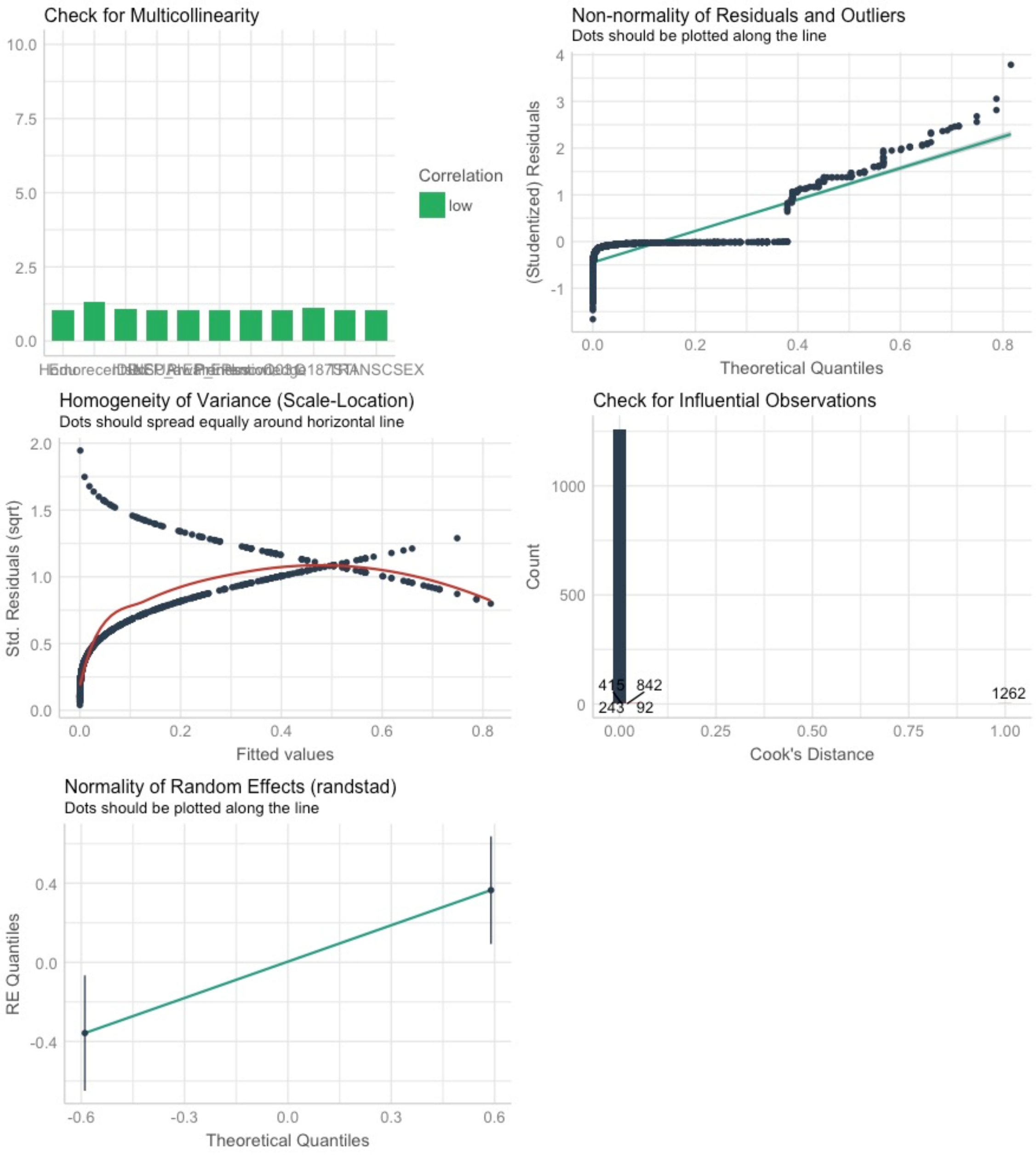

Table S4. Generalised logistic mixed models: variation between regions and the determinants of PrEP uptake, a comparison between the full model and the final model.

	Full model PrEP uptake (AIC = 569.4)			Final model PrEP uptake (AIC = 568.6)		
**Predictors**	**OR**	**95% CI**	**p-value**	**OR**	**95% CI**	**p-value**
(Intercept)	0	0.00 – Inf	0.982	0	0.00 – 0.00	<0.001
PrEP awareness [Yes]	0.35	0.03 – 3.95	0.396	0.4	0.04 – 4.06	0.437
PrEP intention [Yes]	4.61	1.97 – 10.82	<0.001	4.46	1.95 – 10.20	<0.001
Age in years	1	0.98 – 1.03	0.727			
Sexuality [Bisexual]	0.81	0.23 – 2.84	0.739			
Sexuality [Straight or others]	0.5	0.05 – 5.52	0.574			
Disclosure of sexual orientation (outness) [Few or less than half]	0.52	0.22 – 1.21	0.127			
Disclosure of sexual orientation (outness) [More than half]	1.44	0.70 – 2.94	0.318			
Disclosure of sexual orientation (outness) [None]	0	0.00 – Inf	0.993			
Recent gay intercourse sex	1288782.08	0.00 – Inf	0.989			
Decision to have safe sex	1.31	1.02 – 1.69	0.038	1.29	1.03 – 1.61	0.028
Decision to reject sex	0.76	0.56 – 1.03	0.073			
PrEP knowledge	7.66	4.38 – 13.37	<0.001	7.34	4.28 – 12.56	<0.001
HIV knowledge	1.11	0.60 – 2.04	0.746			
PHQ-4 (Depression/ Anxiety status)	1.09	0.79 – 1.51	0.587			
CAGE-4 (Alcohol dependency)	1.33	0.67 – 2.63	0.412			
CAI with non-steady partner [Yes]	1.79	0.85 – 3.80	0.128	2.3	1.11 – 4.77	0.024
CAI with steady partner [Yes]	1.28	0.76 – 2.16	0.346			
CAI with non-steady partner with diagnosed HIV [Yes]	2.16	1.32 – 3.54	0.002	1.97	1.26 – 3.10	0.003
Ever diagnosed of syphilis, gonorrhoea, or chlamydia [Ever]	1.97	1.02 – 3.80	0.042	2.14	1.15 – 3.98	0.016
Transactional sex [Ever but longer than 12 months]	0.47	0.25 – 0.90	0.022	0.52	0.29 – 0.96	0.036
Transactional sex [Never]	0	0.00 – Inf	0.999	0	0.00 – Inf	0.993
Injection drug using [Yes]	2.11	1.03 – 4.33	0.042	2.28	1.20 – 4.34	0.012
Chemsex recency	0.98	0.89 – 1.08	0.724			
Education level [Low]	0.4	0.16 – 0.99	0.046	0.4	0.17 – 0.92	0.03
Education level [Median]	1.32	0.79 – 2.19	0.29	1.24	0.76 – 2.03	0.392
Financial coping [Really struggling]	0.75	0.29 – 1.95	0.557			
Financial coping [Neutral]	0.8	0.45 – 1.40	0.429			
Employment status [Retired]	2.06	0.61 – 6.88	0.242			
Employment status [Student]	0.73	0.25 – 2.14	0.56			
Employment status [Unemployed]	1.39	0.58 – 3.32	0.459			
Random Effects						

σ^2	3.29	3.29
τ_{00}	0.18 $_{randstad}$	0.15 $_{randstad}$
ICC	0.054	0.046
Marginal R^2 / Conditional R^2	0.820 / 0.829	0.706 / 0.719

Note: AIC = Akaike information criterion. OR = odds ratio. 95% CI = 95% confident intervals.
